# The Effects of *Momordica balsamina* Methanolic Extract on Kidney Function in STZ-Induced Diabetic Rats: Effects on Selected Metabolic Markers

**DOI:** 10.1155/2018/7341242

**Published:** 2018-06-13

**Authors:** Anelisiwe Siboto, Ntethelelo Sibiya, Andile Khathi, Phikelelani Ngubane

**Affiliations:** School of Laboratory Medicine and Medical Sciences, University of KwaZulu-Natal, Durban, South Africa

## Abstract

**Background:**

Studies suggest that *Momordica balsamina* (*intshungu*) possesses hypoglycaemic effects. The effects of *Momordica balsamina* on diabetic complications such as DN, however, have not been established. Accordingly, this study seeks to investigate the effects of *M. balsamina* on kidney function in STZ-induced diabetic rats.

**Methods:**

Methanolic extracts (ME) of *M. balsamina*'s leaves were used in this study. Short-term effects of *M. balsamina* methanolic extract on kidney function and MAP were studied in STZ-induced diabetic rats treated twice daily with *M. balsamina* methanolic extract (250 mg/kg), insulin (175 *μ*g/kg, s.c.), and metformin (500 mg/kg) for 5 weeks.

**Results:**

*M. balsamina* methanolic extract significantly increased Na^+^ excretion outputs in STZ-induced diabetic rats by comparison to STZ-diabetic control rats. *M. balsamina* methanolic extract significantly increased GFR in STZ-diabetic rats with a concomitant decrease in creatinine concentration and also reduced kidney-to-body ratio, albumin excretion rate (AER), and albumin creatinine ratio (ACR). *M. balsamina* methanolic extract significantly reduced MAP in STZ-diabetic animals by comparison with STZ-diabetic control animals. These results suggest that *M. balsamina* methanolic extract not only lowers blood glucose but also has beneficial effects on kidney function and blood pressure.

**Conclusion:**

These findings suggest that *M. balsamina* may have beneficial effects on some processes that are associated with renal derangement in STZ-induced diabetic rats.

## 1. Introduction

Diabetic nephropathy (DN), as a result of sustained hyperglycaemia, affects approximately 20–40% of individuals with diabetes mellitus [1]. Diabetic nephropathy is a leading common cause of chronic kidney disease (CKD) and kidney failure which are the major causes of morbidity and mortality associated with type 1 diabetes mellitus [2, 3]. The sustained hyperglycaemia is associated with the structural kidney abnormalities such as the thickening of the glomerular basement membrane, mesangial expansion, tubular hypertrophy, and extracellular matrix deposition [4, 5]. These structural abnormalities mediate the functional kidney abnormalities such as declining glomerular filtration rate, electrolyte imbalance, increased blood pressure, and increased protein excretion [6, 7].

Several mechanisms have been reported to contribute to the development and progression of diabetic nephropathy. These mechanisms include oxidative stress; the advanced glycation end product (AGE) receptor of the AGE pathway and the renin-angiotensin aldosterone system have been implicated to play a role in the development of diabetic nephropathy [8]. These metabolic flux or pathways are mainly activated by sustained hyperglycaemia. Taken together, the activation of these pathways cause the intrarenal haemodynamic's changes through the glycosylation of intrarenal proteins that induce hyperfiltration and glomerular dysfunction [1, 9].

Various agents such as oral antihyperglycaemic drugs, insulin, and antirenin-angiotensin system (RAS) are currently available for the prevention and management of DN; however, the efficacy of these agents is inadequate [10]. Insulin has been shown to normalise glomerular filtration rate (GFR) and oxidative status which helps delay the onset and progression of CKD [11]. However, the bolus administration of insulin in high doses is associated with sodium retention which often leads to the development of hyperinsulinaemic oedema and elevated blood pressure [12]. Other antidiabetic agents such as metformin and insulin secretagogues are at times unable to achieve glycaemic targets and may thus be unable to prevent the progression to DN. Therefore, there is a need to further explore other avenues as means of alternative treatment, thus achieving a holistic therapy for DN.

Medicinal plants have been used for treatment of DM and associated complications [13]. Studies in our laboratory have demonstrated that medicinal plants such as *Syzygium aromaticum* and *Syzygium cordutum* confer renoprotection in streptozotocin- (STZ-) induced diabetic rats [14, 15]. *Mormodica balsamina* (MB), our plant of interest, is fairly common and widespread in southern Africa and is closely related to *M. charantia* [16]. *M. charantia* has been shown to improve renal function by normalising oxidative status [17]. *M. balsamina* has been shown to have hypoglycaemic activity [18]. However, the renoprotective effects of *M. balsamina* have not yet been established. We envisage that evaluation of parameters such as GFR and electrolyte handling will shed some light on the therapeutic effects of *M. balsamina* regarding kidney function. The aim of the study therefore is to investigate the effects of *M. balsamina* on kidney function in STZ-induced diabetic rats.

## 2. Materials and Methods

### 2.1. Drugs and Chemicals

Drugs were sourced from standard pharmaceutical suppliers. All chemicals were of analytical grade and were purchased from standard commercial suppliers.

### 2.2. Plant Extraction

#### 2.2.1. Plant Material

*Momordica balsamina* was identified by a botanist in the Department of Botany at the University of KwaZulu-Natal, Westville. The plant was harvested at the University of KwaZulu-Natal in Durban, South Africa. The leaves were washed three times with water to remove any residual dirt. The leaves were then grinded to a pulp using an electric grinder. Thereafter, the pulp was subjected to either methanolic or aqueous extraction as previously described [18].

#### 2.2.2. Methanolic Extraction (ME)

The pulp (1.15 kg) was extracted by cold percolation with methanol (95%, 6.9 L) for 24 h. The methanolic extract was recovered from the mixture and methanol was further added to the plant material for further extraction. This process was repeated three times to maximise the yield (609 g). The three extracts were pooled together and the combined extract was concentrated at reduced pressure (22–26 mmHg) at 45–60°C.

### 2.3. Animals

Male Sprague-Dawley rats weighing 250–300 g were used to carry out the study. The animals were bred and kept in the Biomedical Research Unit of the University of KwaZulu-Natal under normal laboratory conditions (temperature and humidity) in a 12 h day: 12 h night cycle. The animals were allowed access to water ad libitum and 40 g standard rat chow daily (Meadow Feeds, Pietermaritzburg, South Africa). All animal experiments were reviewed and accepted by the Animal Research Ethics Committee of the University of KwaZulu-Natal (AREC/049/016DM). Before the study commenced, the animals were allowed to acclimatise for 5 days in the metabolic cages.

#### 2.3.1. Diabetes Induction

Type 1 diabetes mellitus was induced in male Sprague-Dawley rats by a single intraperitoneal injection of 60 mg/kg STZ in freshly prepared 0.1 M citrate buffer (pH 6.3). The control group received the vehicle, citrate buffer, through the same route. Seven days after the induction of diabetes, rats with a blood glucose concentration greater than 20 mmol·L^−1^ were considered diabetic.

### 2.4. Experimental Protocol

The short-term effects of *M. balsamina* were studied in separate groups of nondiabetic and STZ-induced diabetic male rats (*n* = 6) housed individually in Makrolon polycarbonate metabolic cages (Techniplast, Labotec, South Africa) for 5 weeks. ME (250 mg/kg, p.o.) was administered twice daily at 0900 h and 1500 h by means of a bulbed steel tube. Animals which received DMSO/saline (3 mL/kg, p.o.) served as controls, while those given metformin (500 mg/kg, p.o.) and insulin (200 *μ*g/kg, s.c.) served as positive controls. Blood glucose measurements were made once every third day over the period of 5 weeks using the tail prick method. Glucose was determined using the Elite® glucometer (Elite (Pty) Ltd., Health Care Division, South Africa). Urine volume and urinary glucose concentrations, albumin, creatinine, urea, Na^+^, K^+^, Cl^−^, body weight, 24-hour water intake, food consumption, and mean arterial pressure (MAP) were determined once every third day. MAP was monitored using a noninvasive tail cuff method with photoelectric sensors (IITC Model 31 Computerised Blood Pressure Monitor, Life Sciences, Woodland Hills, California, USA) as previously described [19]. The unit works with IITC hardware system to measure blood pressure and heart rate in conscious rats. The animals were warmed at ±30°C in an enclosed chamber (IITC Model 31 Computerised Blood Pressure Monitor, Life Sciences, Wood land Hills, California, USA) for 30 minutes before taking blood pressure readings. All measurements were conducted at 0900 h. Creatinine and albumin concentration were measured in plasma and urine samples at the Global Clinical and Viral Laboratory, Amanzimtoti, South Africa. GFR was calculated using a standard formula from measurements of the plasma and urinary concentrations of creatinine and urine flow rate in the fifth week.

### 2.5. Plasma and Tissue Collection

At the end of the 5-week experimental period, all animals were anaesthetised via a gas anaesthetic chamber (100 mg/kg) for 3 minutes using halothane. Blood samples were collected by cardiac puncture into single precooled heparinized containers for biochemical analysis. The blood was centrifuged at 10 ×g in a Hermle Laborechnic GmBH centrifuge (Wehingen, Germany) for plasma collection. The kidneys were removed, weighed, and then snap frozen in liquid nitrogen. Afterwards, the kidneys and plasma were stored in an Ultra Bio Freezer (Snijers Scientific, Tilburg, Netherlands) at −80°C.

### 2.6. Malondialdehyde (MDA) Measurements

MDA measurements were carried out as per a well-established protocol in our laboratory [15]. Briefly, tissues (50 mg) were homogenized in 500 *μ*L of 0.2% phosphoric acid. The homogenate was centrifuged at 400 ×g for 10 min. Subsequently, 400 *μ*L of the homogenate was supplemented with 400 mL 2% phosphoric acid and then separated into two glass tubes, each receiving equal volumes of the solution. Afterwards, 200 *μ*L of 7% phosphoric acid was added into both glass tubes followed by 400 *μ*L of thiobarbituric acid (TBA)/butylated hydroxytoluene (BHT) into one sample test glass tube and 400 *μ*L of 3 mM hydrochloric acid (HCl) into the second glass tube which stood as a blank. Thereafter, 200 *μ*L of 1 M HCl was then added to sample and blank test tubes to establish an acidic pH of 1.5. Both solutions were heated at 100°C for 15 min and allowed to cool to room temperature. 1.5 ML of butanol was then added to the cooled solution; the sample was vortexed for 1 min for rigorous mixing and allowed to settle until two phases could be differentiated. The top butanol layer was transferred to Eppendorf tubes and centrifuged at 13,200 ×g for 6 min. The samples were aliquoted into a 96-well microtiter plate in triplicates, and the absorbance was read at 532 nm using a BioTek mQuant spectrophotometer (Biotek, Johannesburg, South Africa). The absorbance from these wavelengths was used to calculate the concentration of MDA using Beer's law.

(1) $$\begin{array}{c} Concentration=\frac{{absorbance}_{Final}}{absorption\;coefficient\;\left(156\;{mmol}^{-1}\right)}. \end{array}$$

### 2.7. Glutathione Peroxidase (GPx) and Superoxide Dismutase (SOD) Activity

Kidney GPx and SOD activity was measured using assay kits following the manufacturer's instructions (Elabscience and Biotechnology, WuHan).

### 2.8. Aldosterone

Plasma aldosterone was measured using an ELISA kit following the manufacturer's instructions (Elabscience and Biotechnology, WuHan).

### 2.9. Statistical Analysis

Data is expressed as means ± standard error of means (SEM). Statistical analysis was conducted using GraphPad Prism and InStat software (version 5.00, GraphPad Software, San Diego, California, USA). Energy balance parameters, MAP, and urine output were analysed using analysis of variance (ANOVA) for repeated measures, which was followed by the Bonferroni post hoc test. Terminal parameters were analysed using ANOVA followed by the Bonferroni post hoc test, which was used to analyse differences between the controls and the experimental groups. Values of *p* < 0.05 indicate statistical significance.

## 3. Results

### 3.1. Energy Balance

Nondiabetic controls, diabetic controls, and diabetic rats treated with MB, metformin, and insulin were assessed for body weight change and 24-hour water consumption weekly for 5 weeks (Table 1). Diabetic control rats exhibited severe weight loss despite the significantly higher weekly intake of food and water when compared to the normal nondiabetic control rats (DC versus ND, *p* < 0.05). In contrast, nondiabetic control rats progressively gained weight from the 2nd week of treatment until the end of the experimental period. MB-treated (250 mg/kg) STZ-diabetic rats, however, stabilised body weights by comparison to untreated STZ-diabetic controls from the 3rd week to the 5th week of the experimental period while significantly decreasing food intake (*p* < 0.05). The effects of metformin (500 mg/kg) were similar to those of MB. Administration of insulin (175 *μ*g/kg) improved body weight and decreased food and water intake from the 3rd to the 5th week of the experimental period by comparison to untreated STZ-diabetic rats.

### 3.2. Mean Arterial Pressure (MAP)

**Figure 1:**
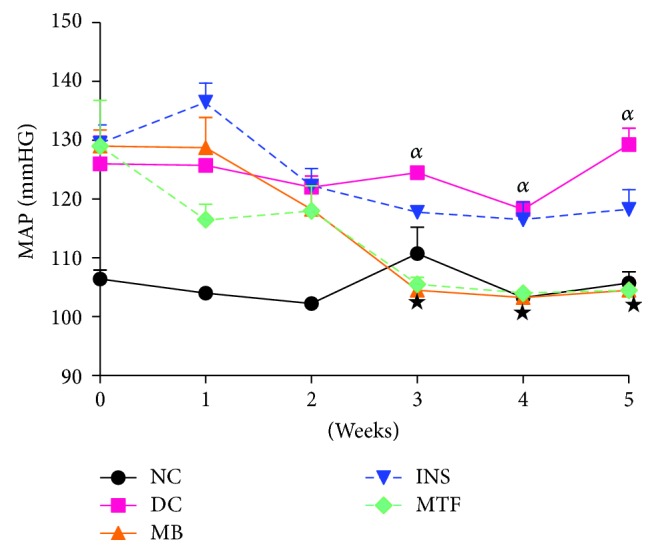
Mean arterial blood pressure of nondiabetic controls, diabetic controls, and diabetic rats treated with *M. balsamina*, metformin, and insulin over a period of 5 weeks. Values are expressed as mean ± SEM. *^α^p* < 0.05 by comparison to nondiabetic control; ^★^*p* < 0.05 by comparison with diabetic control.

Figure 1 shows the mean arterial pressure (MAP) of nondiabetic controls; diabetic controls; and diabetic rats treated with MB, metformin, and insulin measured weekly over the 5-week experimental period. Diabetic control rats showed increased MAP over the period of 5 weeks by comparison to nondiabetic rats. However, the administration of MB stabilised MAP to normalcy from the 3rd week to 5th week by comparison to STZ-diabetic controls. The administration of insulin (175 *μ*g/kg) also increased MAP by comparison to nondiabetic rats. The effects of metformin were similar to MB.

### 3.3. Urine Output

**Figure 2:**
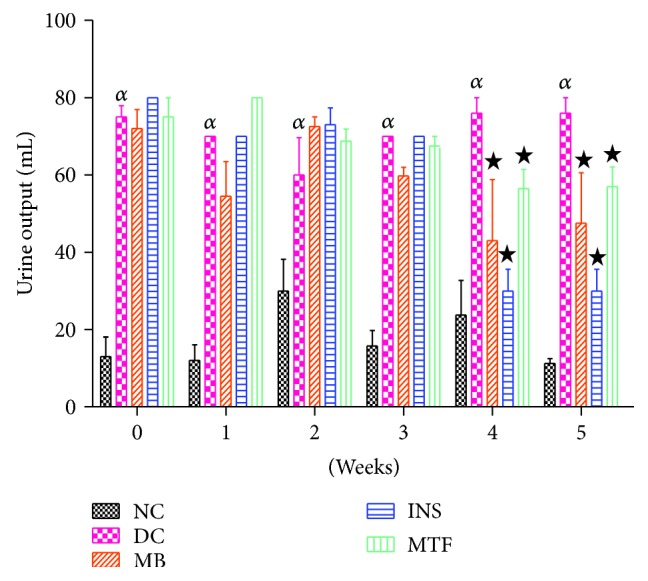
Urine output of nondiabetic controls, diabetic controls, and diabetic rats treated with *M. balsamina*, metformin, and insulin over a period of 5 weeks. Values are expressed as mean ± SEM. *^α^p* < 0.05 by comparison to nondiabetic control; ^★^*p* < 0.05 by comparison with diabetic control.

Figure 2 shows the 24 h urine output of nondiabetic controls, diabetic controls, and diabetic rats treated with MB, metformin, and insulin measured over the 5-week period. Diabetic controls showed increased urine output throughout the study by comparison to nondiabetic rats. The administration of MB (250 mg/kg) decreased urine output from the 4th week to the 5th week of the experimental period by comparison to STZ-diabetic rats. The effects of insulin and metformin were similar to those of MB.

### 3.4. Electrolyte Handling

Table 2 shows urinary and plasma biochemical parameters of nondiabetic controls, diabetic controls, and diabetic rats treated with *M. balsamina*, metformin, and insulin. On the 5th week of the experimental period, plasma creatinine concentrations were significantly (*p* < 0.05) elevated in STZ-diabetic rats in comparison with nondiabetic rats. On the other hand, urinary creatinine concentrations were significantly reduced at the same time point. Administration of *M. balsamina*, however, significantly reduced plasma creatinine concentrations while significantly increasing urinary creatinine concentrations by comparison with STZ-diabetic rats (*p* < 0.05). The effects of metformin and insulin on plasma creatinine and urinary urea were similar to *M. balsamina.* Plasma urea concentrations were significantly (*p* < 0.05) increased in STZ-diabetic rats, while plasma albumin was notably reduced by comparison with nondiabetic control rats. However, treatment with *M. balsamina* significantly (*p* < 0.05) decreased plasma urea concentration while significantly (*p* < 0.05) increasing plasma albumin in comparison with STZ-diabetic control rats. The effects of metformin and insulin on plasma urea and plasma albumin were similar to *M. balsamina*. STZ-diabetic control showed significantly increased plasma Na^+^ concentration, aldosterone and decreased urinary Na^+^ concentrations at week 5 by comparison with nondiabetic rats (*p* < 0.05). Administration of *M. balsamina*, however, significantly (*p* < 0.05) reduced plasma Na^+^ and aldosterone concentrations, while urinary Na^+^ concentrations significantly (*p* < 0.05) increased at week 5 by comparison to STZ-induced diabetic rats. The effects of metformin were similar to *M. balsamina*. Insulin administration increased plasma Na^+^ and aldosterone concentrations while urinary Na^+^ was decreased. Urinary K^+^ concentrations were significantly (*p* < 0.05) decreased, while plasma K^+^ concentrations were significantly (*p* < 0.05) elevated in STZ-diabetic rats by comparison to nondiabetic rats. Administration of *M. balsamina* significantly (*p* < 0.05) increased urinary K^+^ output and decreased plasma K^+^ concentrations on the 5th week of experimental period. The effects of *M. balsamina* on urinary K^+^ output and plasma K^+^ were comparable with those of metformin and insulin.

### 3.5. Kidney Weights

Table 3 shows kidney-to-body weight ratio of nondiabetic controls, diabetic controls, and diabetic rats treated with *M. balsamina*, metformin, and insulin measured after 5 weeks of treatment. The kidney-to-body weight ratio was significantly increased in STZ-diabetic rats by comparison to nondiabetic controls (*p* < 0.05). The administration of MB, insulin, or metformin did not affect the kidney-tobody weight ratio by comparison to STZ-diabetic controls.

### 3.6. Kidney Function

Table 4 shows factors of kidney function of nondiabetic controls, diabetic controls, and diabetic rats treated with *M. balsamina*, metformin, and insulin assessed after the 5-week experimental period. STZ-diabetic control animals showed significantly increased AER, ACR, and GFR in comparison with nondiabetic animals (*p* < 0.05). The administration of *M. balsamina*, however, significantly decreased AER, ACR, and GFR on the 5th week of experimental period (*p* < 0.05). The effects of insulin were similar to those of MB.

### 3.7. Oxidative Stress Markers

Table 5 shows kidney malondiadehyde (MDA) concentration and activities of SOD and GPx in nondiabetic controls; diabetic controls; diabetic rats treated with MB, metformin, and insulin measured after 5 weeks of experimental period. Diabetic control rats had significantly increased kidney MDA concentration and had a decrease activity of SOD and GPx in comparison with nondiabetic control (*p* < 0.05). STZ-diabetic rats treated with MB, insulin (175 *μ*g/kg) or metformin (500 mg/kg) showed significantly decreased MDA levels and increased activity of SOD and GPx in kidneys by comparison with STZ-induced diabetic control rats (*p* < 0.05).

## 4. Discussion

The current study investigated the effects of *M. balsamina* on renal function in STZ-induced diabetic rats. The results herein indicate that *M. balsamina* is effective in ameliorating renal fluid and electrolyte handling in STZ-induced diabetic rats. In addition, *M. balsamina* reduces oxidative stress and MAP in STZ-induced diabetic rats.

Diabetes is a metabolic disorder characterised by hyperglycaemia. Previous studies, however, have reported that medicinal plants have hypoglycaemic effects [14, 20]. In agreement with previous reports, the use of *M. balsamina* crude extract in STZ-induced diabetic rats significantly attenuated blood glucose concentration in this study. Diabetes mellitus is also associated with polyphagia, polydipsia, and reduction in body weight [21]. Treatment with *M. balsamina*, however, lowered food intake while improving body weight.

Sustained hyperglycaemia has been shown to activate the intrarenal renin aldosterone system which is thought to increase Na^+^ retention at various sites of the kidney such as distal tubule and collecting duct of the nephron [22]. Na^+^ retention, as assisted by increased aldosterone levels, has been shown to increase MAP in STZ-diabetic rats as also seen in this study [23]. *M. balsamina*, however, decreased MAP of STZ-induced diabetic rats possibly via the inhibition of intrarenal renin aldosterone system as *M. balsamina* administration resulted in decreased concentrations of plasma aldosterone in comparison to untreated STZ-diabetic rats [22, 23]. The results are of importance because diabetes is well known to lead to high blood pressure that eventually results in cardiovascular complications [24].

STZ-induced diabetes is associated with increased kidney weights, albumin excretion rate (AER), and albumin creatinine ratio (ACR) due to the accumulation of extracellular proteins and kidney damage [25]. Treatment with *M. balsamina*, however, attenuated kidney hypertrophy as evidenced by the reduced kidney to body ratio, AER, and ACR. The extracellular protein-induced kidney hypertrophy has been shown to cause structural changes such as the thickening of the glomerular basement membrane, thereby impairing kidney function which includes fluid and electrolyte imbalance in acute renal failure [25]. Furthermore, the impairment of kidney function as a result of the thickening of the glomerular basement membrane is also associated with elevated creatinine concentration with a reduction in GFR as seen in the STZ-diabetic rats in this study at the end of the experimental period. These changes, however, were attenuated in the *M. balsamina*-treated animals as *M. balsamina* increased urinary Na^+^ outputs of STZ-diabetic rats and elevated GFR as assessed by creatinine clearance, suggesting an upregulation of renal function. GFR is an important marker of kidney function, and treatment-related increases in creatinine clearance indicate improvement of kidney function in experimental animals [11]. In addition, increases in AER and ACR are markers of the thickening basement membrane [25].

The significant increase in the excretion of Na^+^ by *M. balsamina* may perhaps occur via the inhibition of increased proximal reabsorption of Na^+^ which is often seen in DM [26]. Kidneys maintain the optimum chemical composition of body fluids by removing Na^+^, K^+^, urea, albumin, and creatinine [27, 28]. The concentration of these electrolytes, however, was increased in the plasma of STZ-diabetic animals possibly due to sustained hyperglycaemia [28]. Treatment with *M. balsamina*, however, was shown to increase excretion of urea and albumin in STZ-diabetic rats possibly via the amelioration of hyperglycaemia. Studies have shown that improved glycaemic control is beneficial in improving kidney function [28].

Hyperglycaemia-induced oxidative stress in diabetes is a major cause for the development and progression of diabetic microvascular complications such as diabetic nephropathy [29]. In diabetes mellitus, hyperglycaemia can simply inactivate antioxidant enzymes such as SOD and GPx by glycating these proteins and inducing oxidative stress which in turn causes lipid peroxidation [30]. Decreased antioxidant enzyme levels and enhanced lipid peroxidation have been well documented in STZ-induced diabetes [31]. In the enzymatic antioxidant defence system, SOD is one of the important enzymes which scavenge the superoxide radicals by converting them to H_2_O_2_ and molecular oxygen [8, 30]. The observed decrease in SOD activity in diabetic control rats could result from inactivation by H_2_O_2_ or by glycosylation of the enzyme, which has been reported to occur in diabetes [30]. GPx is also involved in the elimination of H_2_O_2_ [32, 33]. The decreased activity of SOD and GPx in the diabetic state may be due to inactivation caused by reactive oxygen species. The results herein showed that elevated concentrations of MDA, a marker of lipid peroxidation, were restored after 5 weeks of treatment with *M. balsamina*. The reduction of MDA levels could be due to improvement in glycaemic control and also increased antioxidant status since *M. balsamina* significantly stimulated the increased activity of SOD and GPx. Medicinal plant extracts have been shown to have antioxidant properties due to their phytochemicals such as alkaloids, polyphenols, saponins, vitamins (C and E), carotenoids, and flavonoids [13–15]. All these have protective roles against oxidative damage, thus preventing lipid peroxidation and protecting the kidneys from severe increases of reactive oxygen species and depletion of superoxide dismutase and reduced glutathione [15, 34, 35]. Studies in our laboratory have indeed shown that reduction of blood glucose may prevent oxidative stress and therefore delay the onset of diabetic complications [14, 15]. Taken together, these results suggest that *M. balsamina* protects the kidneys from damage that occurs in the STZ-induced diabetic animal model.

## 5. Conclusion

The results described in this study demonstrated that *M. balsamina* ameliorates kidney dysfunction associated with diabetes as shown by increased urinary Na^+^ outputs and reduction of plasma creatinine with concomitant increase in GFR. These observations warrant further investigations on *M. balsamina* as a potential alternative remedy for diabetes-associated kidney dysfunction.

## Figures and Tables

**Table 1:** Comparison of food, water intake, and body weight (b.wt) change of nondiabetic controls, diabetic controls, and diabetic rats treated with MB, metformin, and insulin during a 5-week study (*n* = 6 in each group). Values are expressed as mean ± SEM.

Parameter	Treatment	Time (weeks)				
		1	2	3	4	5
Food intake (g/100 g)	NC	11.9 ± 3.4	12.2 ± 1.6	12.3 ± 1.7	13.0 ± 0.4	12.9 ± 0.9
	DC	27.6 ± 1.2	25.4 ± 1.4	16.7 ± 0.4	28.0 ± 1.1	24.8 ± 0.3
	MB	10.0 ± 2.1^∗^	10.2 ± 2.6^∗^	16.4 ± 1.1	26.47 ± 2.4^∗^	18.9 ± 0.6
	INS	19.2 ± 2.2^∗^	18.8 ± 1.9^∗^	11.2 ± 0.8	15.5 ± 3.0^∗^	14.2 ± 0.9^∗^
	MTF	28.2 ± 0.6^∗^	22.7 ± 0.5	21.8 ± 0.5	19.8 ± 0.7	26.8 ± 1.7

Water intake (mL/100 g)	NC	30 ± 8.2	35 ± 1.4	32.5 ± 8.5	45 ± 9.6	51.2 ± 7.2
	DC	60 ± 9.6	61.3 ± 12.9	95.0 ± 3.7	91.3 ± 3.4	96 ± 1.0
	MB	72.5 ± 2.5	67.0 ± 4.1	73.8 ± 8.3	78.7 ± 9.6	76 ± 1.0
	INS	73.0 ± 4.4	76.3 ± 9.4	76.3 ± 0.1	76.4 ± 4.8	74.2 ± 2.3
	MTF	68.8 ± 3.1	41.3 ± 7.5	45.6 ± 2.5	45.2 ± 2.3	43.8 ± 1.1

% b.wt change/week	NC	44.7 ± 9.4	69.7 ± 5.9	76 ± 8.6	90.7 ± 11.1	92.5 ± 9.9
	DC	−72 ± 8.6	−87.2 ± 20	−77.5 ± 9.8	−77.5 ± 12	−80 ± 7.4
	MB	−13.8 ± 8^∗^	−11.1 ± 5^∗^	−19.5 ± 6.2^∗^	−15.3 ± 6.1^∗^	−40.5 ± 11
	INS	18.5 ± 13.9^∗^	3.2 ± 15.7^∗^	30.0 ± 16.2^∗^	38.3 ± 18.1^∗^	28.3 ± 3.2^∗^
	MTF	−23 ± 7.3	−6 ± 6.8	−32 ± 2.9	−11 ± 17.3	−37 ± 3.2

Glucose (mmol/L)	NC	5.1 ± 0.3	5.1 ± 0.4	4.9 ± 0.3	5.8 ± 0.5	5.3 ± 0.1
	DC	33.1 ± 1.5^#^	33.1 ± 0^#^	33.4 ± 0.2^#^	33.0 ± 0.8^#^	33.2 ± 0.2^#^
	MB	27.2 ± 0.3	29.1 ± 0.3	24.2 ± 1.9^∗^	25.6 ± 0.6^∗^	26.4 ± 1.0^∗^
	INS	30.7 ± 0.8	23.2 ± 2.6	21.1 ± 2.3^∗^	21.9 ± 1.6^∗^	17.1 ± 0.9^∗^
	MTF	28.0 ± 2.1	26.0 ± 1.4	25.7 ± 0.5^∗^	24.6 ± 0.9^∗^	24.8 ± 0.6^∗^

^#^ *p* < 0.05 by comparison with nondiabetic control and ^∗^*p* < 0.05 by comparison with diabetic control.

**Table 2:** Comparison of plasma biochemical parameters and urinary electrolytes of nondiabetic controls, diabetic controls, and diabetic rats treated with MB, metformin, and insulin during a 5-week study (*n* = 6 in each group). Values are expressed as mean ± SEM.

Parameters	Groups				
	NC	DC	MB	INS	MTF
Urinary Na^+^ (mmol/L)	66.8 ± 1.2	42.3 ± 3.5^#^	53.7 ± 1.3^∗^	38.2 ± 1.2	66.0 ± 2.9^∗^
Plasma Na^+^ (mmol/L)	120.3 ± 3.3	134.0 ± 2.0^#^	121.5 ± 4.2^∗^	133.2 ± 1.3	121.7 ± 1.4^∗^
Urinary K^+^ (mmol/L)	45.5 ± 1.7	25.5 ± 2.1^#^	53.7 ± 1.3^∗^	38.2 ± 3.3^∗^	55.5 ± 4.1^∗^
Plasma K^+^ (mmol/L)	9.9 ± 1.3	7.6 ± 0.6^#^	7.9 ± 0.3	9.5 ± 0.7^∗^	8.4 ± 0.4^∗^
Urinary urea (mmol/L)	654.3 ± 6.8	117.8 ± 4.8^#^	315.3 ± 5.8^∗^	356 ± 9.9^∗^	351 ± 8.0^∗^
Plasma urea (mmol/L)	4.9 ± 0.3	17.5 ± 1.3^#^	7.0 ± 0.6^∗^	7.6 ± 0.8^∗^	6.4 ± 0.9^∗^
Urinary albumin (g/L)	1.7 ± 0.2	3.5 ± 0.4^#^	3.2 ± 0.7	4.2 ± 0.7^∗^	4.7 ± 1.1^∗^
Plasma albumin (g/L)	16.5 ± 1.0	12.3 ± 0.6^#^	13.2 ± 0,6	14.3 ± 0.7^∗^	14.3 ± 1.3^∗^
Urinary creatinine (*μ*mol/L)	9.9 ± 0.7	14.9 ± 0.3^#^	10.4 ± 0.2^∗^	8.1 ± 0.4^∗^	10.8 ± 0.2^∗^
Creatinine (*μ*mol/L)	32.5 ± 1.3	45.8 ± 1.1^#^	29.8 ± 1.0^∗^	34.8 ± 1.9^∗^	28.2 ± 0.8^∗^
Aldosterone (pg/mL)	0.2 ± 0.02	0.4 ± 0.1^#^	0.3 ± 0.4^∗^	0.4 ± 0.2	0.2 ± 0.1^∗^

^#^ *p* < 0.05 by comparison with nondiabetic control; ^∗^*p* < 0.05 by comparison with diabetic control.

**Table 3:** Effects of *M. balsamina* on the kidney-to-body weight ratio after a 5-week study (*n* = 6 in each group). Values are expressed as mean ± SEM.

Experimental groups	Final body weight (g)	Kidney weight (g)	Kidney : body weight ratio (%)
NC	342.5 ± 9.92	1.11 ± 0.081	0.32 ± 0.02
DC	170 ± 7.39^#^	1.37 ± 0.09^#^	0.80 ± 0.02^#^
MB	209 ± 11.33^∗^	1.01 ± 0.031^∗^	0.53 ± 0.03^∗^
INS	278.5 ± 7.5^∗^	1.14 ± 0.14^∗^	0.41 ± 0.06^∗^
MTF	212.5 ± 0.04^∗^	0.97 ± 0.40^∗^	0.46 ± 0.023^∗^

^#^ *p* < 0.05 by comparison with nondiabetic control; ^∗^*p* < 0.05 by comparison with diabetic control.

**Table 4:** Comparison albumin excretion rate (AER), albumin creatinine ratio (ACR), and glomerular filtration rate (GFR) of nondiabetic controls, diabetic controls, and diabetic rats treated with MB, metformin, and insulin rats after a 5-week study (*n* = 6 in each group). Values are expressed as mean ± SEM.

Parameters	Groups				
	NC	DC	MB	INS	MTF
AER (mg/day)	0.024 ± 0.01	0.29 ± 0.03^#^	0.17 ± 0.01^∗^	0.15 ± 0.01^∗^	0.19 ± 0.03^∗^
ACR	8.10 ± 013	27.03 ± 1.13^#^	15.72 ± 1.50^∗^	13.48 ± 0.84^∗^	19.23 ± 0.28^∗^
GFR (mL/min/100 g)	33.5 ± 0.5	24.8 ± 1.3^#^	33.7 ± 1.5^∗^	37.9 ± 1.3^∗^	30.9 ± 2.1^∗^

^#^ *p* < 0.05 by comparison with nondiabetic control; ^∗^*p* < 0.05 by comparison with diabetic control.

**Table 5:** Effects of MB on MDA concentrations and activities of SOD and GPx in kidney tissues of non-diabetic controls, diabetic controls, and diabetic rats treated with MB, metformin, and insulin. Values are expressed as mean ± SEM.

Parameter measured	Treatment	Kidney
MDA (nmol·g^−1^ protein)	NC	0.994 ± 0.020
	DC	4.21 ± 0.12^#^
	INS	1.1 ± 0.01^∗^
	MTF	1.19 ± 0.01^∗^
	MB	1.14 ± 0.01^∗^

SOD activity (pg/mL)	NC	13.43 ± 0.76
	DC	6.01 ± 0.09^#^
	INS	9.89 ± 0.10^∗^
	MTF	10.11 ± 0.02^∗^
	MB	10.08 ± 0.05^∗^

GPx activity (ng/mL)	NC	2137.31 ± 0.03
	DC	1703.491 ± 0.03^#^
	INS	2062.21 ± 0.02^∗^
	MTF	2125.75 ± 0.01^∗^
	MB	21370.37 ± 0.03^∗^

^#^ *p* < 0.05 by comparison with nondiabetic control; ^∗^*p* < 0.05 by comparison with diabetic control.
